# Changes in the Content of Organic Acids and Expression Analysis of Citric Acid Accumulation-Related Genes during Fruit Development of Yellow (*Passiflora edulis* f. *flavicarpa*) and Purple (*Passiflora edulis* f. *edulis*) Passion Fruits

**DOI:** 10.3390/ijms22115765

**Published:** 2021-05-28

**Authors:** Xiaoxue Zhang, Xiaoxia Wei, Muhammad Moaaz Ali, Hafiz Muhammad Rizwan, Binqi Li, Han Li, Kaijie Jia, Xuelian Yang, Songfeng Ma, Shaojia Li, Faxing Chen

**Affiliations:** 1 College of Horticulture, Fujian Agriculture and Forestry University, Fuzhou 350002, China; 13647152752@139.com (X.Z.); muhammadmoaazali@yahoo.com (M.M.A.); chrizwan51@gmail.com (H.M.R.); libinqi2020@126.com (B.L.); lh138158@126.com (H.L.); jkj2021525@126.com (K.J.); yangxuelian1995@163.com (X.Y.); masongfeng2008@163.com (S.M.); 2 Fruit Research Institute, Fujian Academy of Agricultural Sciences, Fuzhou 350002, China; zhw7782352@sina.com; 3 College of Agriculture and Biotechnology, Zhejiang University, Hangzhou 310058, China

**Keywords:** fruit quality, sugar-acid ratio, fruit ripening, PEPC, cyt-ACO, UPLC, qRT-PCR, *Passiflora edulis* Sims, isocitrate dehydrogenase

## Abstract

Organic acids are key components that determine the taste and flavor of fruits and play a vital role in maintaining fruit quality and nutritive value. In this study, the fruits of two cultivars of passion fruit Yellow (*Passiflora edulis* f. *flavicarpa*) and purple (*Passiflora edulis* f. *edulis*) were harvested at five different developmental stages (i.e., fruitlet, green, veraison, near-mature and mature stage) from an orchard located in subtropical region of Fujian Province, China. The contents of six organic acids were quantified using ultra-performance liquid chromatography (UPLC), activities of citric acid related enzymes were determined, and expression levels of genes involved in citric acid metabolism were measured by quantitative real-time PCR (qRT-PCR). The results revealed that citric acid was the predominant organic acid in both cultivars during fruit development. The highest citric acid contents were observed in both cultivars at green stage, which were reduced with fruit maturity. Correlation analysis showed that citrate synthase (CS), cytosolic aconitase (Cyt-ACO) and cytosolic isocitrate dehydrogenase (Cyt-IDH) may be involved in regulating citric acid biosynthesis. Meanwhile, the *PeCS2, PeACO4, PeACO5* and *PeIDH1* genes may play an important role in regulating the accumulation of citric acid. This study provides new insights for future elucidation of key mechanisms regulating organic acid biosynthesis in passion fruit.

## 1. Introduction

The passion fruit is native to tropical America and has more than 500 species of which at least 50 or more are edible [1]. At present, *Passiflora edulis* f. *flavicarpa* and *Passiflora edulis* f. *edulis* are the main cultivated varieties, widely appreciated and accepted by consumers worldwide due to its unique flavor and high medicinal value [2,3]. Organic acids, being the important constituent of fruit taste along with sugars, strongly influence the organoleptic quality of fruits [4,5]. The sugar–acid ratio is one of the important indicators to measure the flavor and maturation of fruit and is mainly affected by organic acid contents [6,7,8,9]. Many studies have been reported to ascertain compositional changes in passion fruit, and most of these studies have focused on the volatile compounds [10,11,12].

A moderate quantity of acids can make the fruit more palatable, but higher acid contents often reduce fruit quality. Organic acids are accumulated during fruit growth and are consumed as respiratory substrates with fruit ripening. The final organic acid concentration in ripened fruits depends on the balance of organic acid synthesis, membrane transport and degradation or utilization [13,14]. In this process, acid metabolism-related enzymes including citrate synthase (CS), phosphoenolpyruvate carboxylase (PEPC), aconitase (ACO), and isocitrate dehydrogenase (IDH) may potentially play a role in fruit organic acid biosynthesis and degradation [15]. Citric acid synthesis initiates with phosphoenolpyruvate (PEP) in the cytosol, which moves actively into the mitochondrion where it is converted to oxaloacetate (OAA) by the catalyzation of phosphoenolpyruvate carboxylase (PEPC). Then, citric acid is synthesized by the condensation of acetyl-CoA and OAA catalyzed by citrate synthase (CS). Subsequently, citrate can be degraded to α-ketoglutarate (α-KG) through the activities of aconitase (ACO) and NADP-dependent isocitrate dehydrogenase (NADP-IDH) in the cytosol [16]. These metabolic pathways indicate that CS and PEPC mainly catalyze the synthesis of citric acid, while ACO and IDH promote the decomposition of citric acid [17].

Although the contents and components of organic acid in passion fruits have been investigated at mature stage in some details [18,19], dynamics of organic acids contents, enzymatic activity and related gene expression during passion fruit development are still unclear. In the present study, changes of organic acid contents in two cultivars of passion fruit at five developmental stages were studied, and then, the activity of crucial enzymes involved in organic acid metabolism and the expression of the related genes was analyzed. Our findings will provide useful information for improving the organoleptic quality of passion fruit via reducing the contents of citric acid.

## 2. Results

### 2.1. Fruit Weight, Total Soluble Solids, Titratable Acidity and Sugar-Acid Ratio

**Figure 1:**
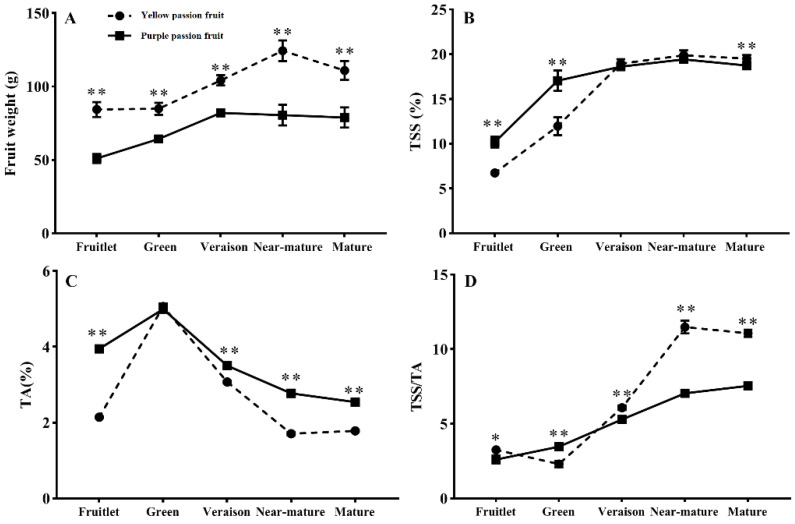
Changes in fruit weight (**A**), total soluble solids (TSS) (**B**), titratable acidity (TA) (**C**) and sugar-acid ratio (**D**) of two passion fruit cultivars (i.e., ‘yellow passion fruit’ and ‘purple passion fruit’) during fruit growth and development. Vertical bars indicate means ± SD (*n* = 15). The * and ** represent significance at *p* ≤ 0.05 and *p* ≤ 0.01, respectively, according to Student’s *t*-test.

The fresh weight (FW) of ‘yellow passion fruit’ showed a typical single sigmoid growth curve, whereas in the case of ‘purple passion fruit’, it increased rapidly from the fruitlet stage to the veraison stage and then slightly declined at the mature stage. In yellow and purple passion fruits, the highest FW was achieved at near-mature stage and veraison stage, respectively. This indicated that the yellow cultivar had a longer growth cycle than the purple one. Moreover, FW of the ‘yellow passion fruit’ was significantly higher than that of ‘purple passion fruit’ during all development stages (Figure 1A). The total soluble solids (TSS) of both cultivars continuously increased from the fruitlet stage to near-mature stage and then slightly decreased toward maturation. At commercial harvesting stage (veraison), there was no significant difference (*p* ≤ 0.05) in TSS of both passion fruit cultivars. However, TSS of ‘yellow passion fruit’ were significantly higher than that of ‘purple passion fruit’ at mature stage (Figure 1B). Total titratable acidity (TA) of both cultivars (yellow and purple passion fruit) increased and reached a peak at green stage with 5.06 and 4.99% of citric acid per 100 g juice, respectively. Then, TA rapidly decreased from green stage to near-mature stage and slightly decreased during maturation. Except for the green fruit period, TA of ‘purple passion fruit’ was significantly higher (*p* ≤ 0.05) than that of ‘yellow passion fruit’ throughout fruit development (Figure 1C). The sugar–acid ratio (TSS/TA) of both cultivars generally exhibited an increasing trend during fruit development, while TSS/TA of ‘yellow passion fruit’ rose significantly faster than that of ‘purple passion fruit’ in later developmental stages (Figure 1D).

### 2.2. Organic Acids

**Figure 2:**
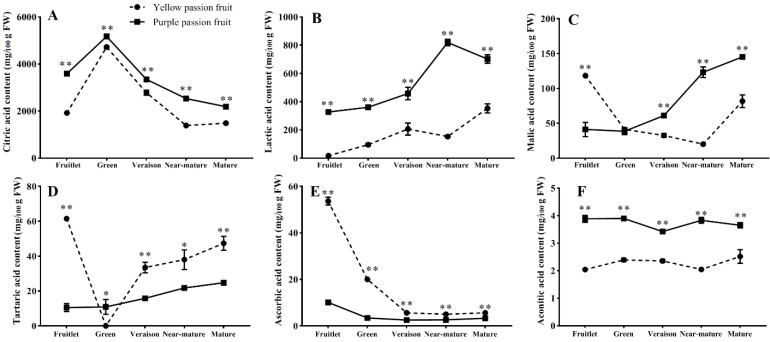
Changes in citric acid (**A**), lactic acid (**B**), malic acid (**C**), tartaric acid (**D**), ascorbic acid (**E**) and aconitic acid (**F**) of two passion fruit cultivars (i.e., ‘yellow passion fruit’ and ‘purple passion fruit’) during fruit growth and development. Vertical bars indicate means ± SD (*n* = 3). The * and ** represent significance at *p* ≤ 0.05 and *p* ≤ 0.01, respectively, according to Student’s *t*-test.

Six organic acids (i.e., citric acid, malic acid, lactic acid, tartaric acid, ascorbic acid and aconitic acid) in yellow and purple passion fruits were detected by UPLC (Figure 2). The results indicated that citric acid was the most abundant organic acid followed by lactic acid. The smaller concentrations of malic acid, tartaric acid, ascorbic acid and aconitic acid were also detected in fruit pulp of passion fruit. Both cultivars exhibited similar pattern of citric acid accumulation throughout fruit development. Citric acid contents of yellow and purple passion fruits increased from the fruitlet stage to green stage and reached the highest peaks (4720.95 and 5170.87 mg·100 g^−1^ FW, respectively) and then sharply decreased as fruit developed. The citric acid content of ‘purple passion fruit’ was significantly (*p* ≤ 0.05) greater than that of ‘yellow passion fruit’ throughout fruit development (Figure 2A).

The lactic acid contents of both cultivars had the same tendency except ‘yellow passion fruit’ showed relatively low level of acid at near-mature stage as compared to the trend in ‘purple passion fruit’. Fruits of both cultivars showed an ultimate increase in acid accumulation during fruit development (Figure 2B). However, the patterns of change in malic acid and tartaric acid differed greatly between the two cultivars. Malic and tartaric acid contents of ‘yellow passion fruit’ decreased from fruitlet stage to green or near-mature stage and then increased quickly until maturity, while in ‘purple passion fruit’ they kept increasing steadily, reaching the highest value in the fruit ripening period (Figure 2C,D). Ascorbic acid content of both cultivars was very high and significantly different (*p* ≤ 0.05) at early growth stage (fruitlet) (53.64 mg·100 g^−1^ FW ‘yellow passion fruit’, 10.15 mg·100 g^−1^ FW ‘purple passion fruit’). Thereafter, this content declined and remained almost constant at low levels for both cultivars during maturation (Figure 2E). The changes in aconitic acid contents of both cultivars remained non-significant from the fruitlet stage to mature stage, with slight changes during development (Figure 2F).

### 2.3. Key Enzymes Involved in Citric Acid Metabolism

**Figure 3:**
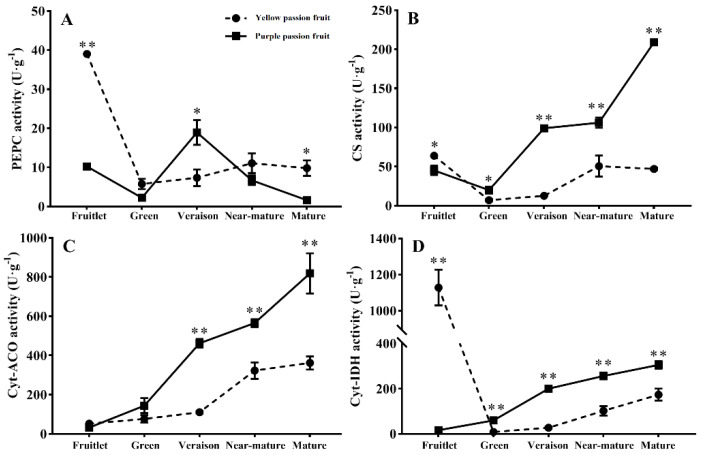
Changes in phosphoenolpyruvate carboxylase (PEPC) (**A**), citrate synthase (CS) (**B**), cytosolic aconitase (Cyt-ACO) (**C**) and cytosolic isocitrate dehydrogenase (Cyt-IDH) (**D**) activities of two passion fruit cultivars (i.e., ‘yellow passion fruit’ and ‘purple passion fruit’) during fruit growth and development. Vertical bars indicate means ± SD (*n* = 3). The * and ** represent significance at *p* ≤ 0.05 and *p* ≤ 0.01, respectively, according to Student’s *t*-test.

In general, both cultivars exhibited remarkable decrease in PEPC activity from the fruitlet stage to green stage. In specific, the PEPC activity in ‘yellow passion fruit’ increased with fruit development and slightly dropped at mature stage, whereas the enzyme’s activity in ‘purple passion fruit’ increased rapidly to a high level at veraison stage and then decreased quickly to the lowest value at mature stage (Figure 3A). Both cultivars exhibited similar pattern of change in CS activity during fruit development (decreasing from the fruitlet stage to green stage, increasing until near-mature stage). However, the CS activity in ‘purple passion fruit’ increased sharply toward fruit maturation, whereas the enzyme’s activity in ‘yellow passion fruit’ decreased slightly. In addition, the CS activity in ‘purple passion fruit’ was significantly higher than that in ‘yellow passion fruit’ from green stage to mature stage (Figure 3B).

The Cyt-Aco activity was relatively low at early growth stage in both cultivars. These activities dramatically increased and remained constant at relatively high levels from the green stage to mature stage and were accompanied with the reduction in citric acid content. Moreover, the Cyt-Aco activity in ‘purple passion fruit’ was significantly higher than that in ‘yellow passion fruit’ at the late stage of fruit development (Figure 3C). The Cyt-IDH activity in ‘yellow passion fruit’ decreased rapidly from the fruitlet stage to green stage, then increased gradually with fruit development. In contrast, in ‘purple passion fruit’ fruits, the Cyt-IDH activity was 15.98 unit g^−1^ FW at fruitlet stage and then increased rapidly to 305.13 unit g^−1^ FW at mature stage. Furthermore, the Cyt-IDH activity in ‘purple passion fruit’ was significantly higher than that in ‘yellow passion fruit’ at the late stage of fruit development (Figure 3D).

### 2.4. Expression Analysis of Genes Encoding Key Enzymes for Citric Acid Metabolism

**Figure 4:**
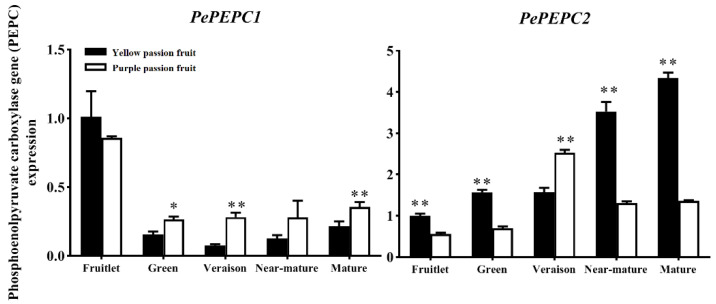
Relative expression of *PePEPC* genes in two passion fruit cultivars, (i.e., ‘yellow passion fruit’ and ‘purple passion fruit’) during fruit growth and development. Vertical bars indicate means ± SD (*n* = 3). The * and ** represent significance at *p* ≤ 0.05 and *p* ≤ 0.01, respectively, according to Student’s *t*-test.

Passion fruits of both cultivars showed more quantity of citric acid as compared to other organic acids (Figure 4), suggesting that citric acid is the main organic acid responsible for the differences in taste of passion fruits. The expression levels of genes involved in citric acid metabolism during fruit growth and development were analyzed based on the transcriptome sequencing results and homologous sequences in the NCBI database (https://www.ncbi.nlm.nih.gov/, accessed on 15 August 2020).

The citrate biosynthesis is severely influenced by PEPC, as it catalyzes the β-carboxylation of PEP to produce oxaloacetic acid (OAA) and inorganic phosphoric acid [20]. The passion fruit transcriptome databases were screened, and the PCR confirmation indicated that there were at least two PEPC gene members in the passion fruit genome databases. The expression level of *PEPC*1 gene in both cultivars decreased rapidly from the fruitlet stage to green stage, followed by the same gene remained relatively constant at the low level until maturity (Figure 4). In contrast, The *PEPC*2 gene of both cultivars maintained a low level in the early stage of fruit development and then gradually increased when the fruits were close to maturity. In addition, the *PEPC1* transcript levels were significantly lower in ‘yellow passion fruit’ than ‘purple passion fruit’ during most sampling periods, whereas the *PEPC2* transcript level in ‘yellow passion fruit’ was markedly higher than that in ‘purple passion fruit’.

**Figure 5:**
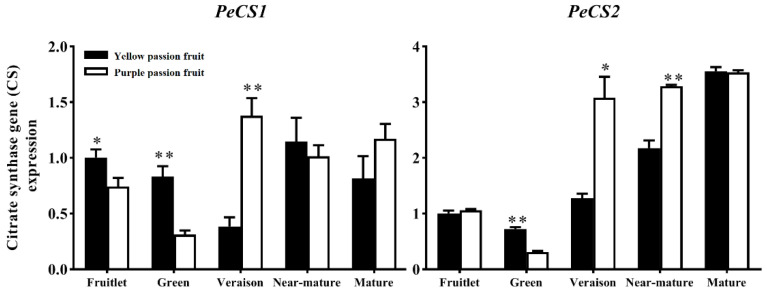
Relative expression of *PeCS* genes in two passion fruit cultivars, (i.e., ‘yellow passion fruit’ and ‘purple passion fruit’) during fruit growth and development. Vertical bars indicate means ± SD (*n* = 3). The * and ** represent significance at *p* ≤ 0.05 and *p* ≤ 0.01, respectively, according to Student’s *t*-test.

Citrate synthase (CS) in involved in citrate accumulation by catalyzing the condensation of acetyl-CoA and OAA [21]. The sequence determination and PCR validation suggested that at least two genes encoding CS exist in the passion fruit genome. The *CS1* and *CS2* genes in both cultivars showed a trend of initially decreasing and then increasing throughout fruit development which coincided with the change of CS enzyme activity (Figure 5). However, the *CSs* transcript levels were significantly higher in ‘yellow passion fruit’ than ‘purple passion fruit’ at the beginning of fruit development, while vice versa at the later stage.

**Figure 6:**
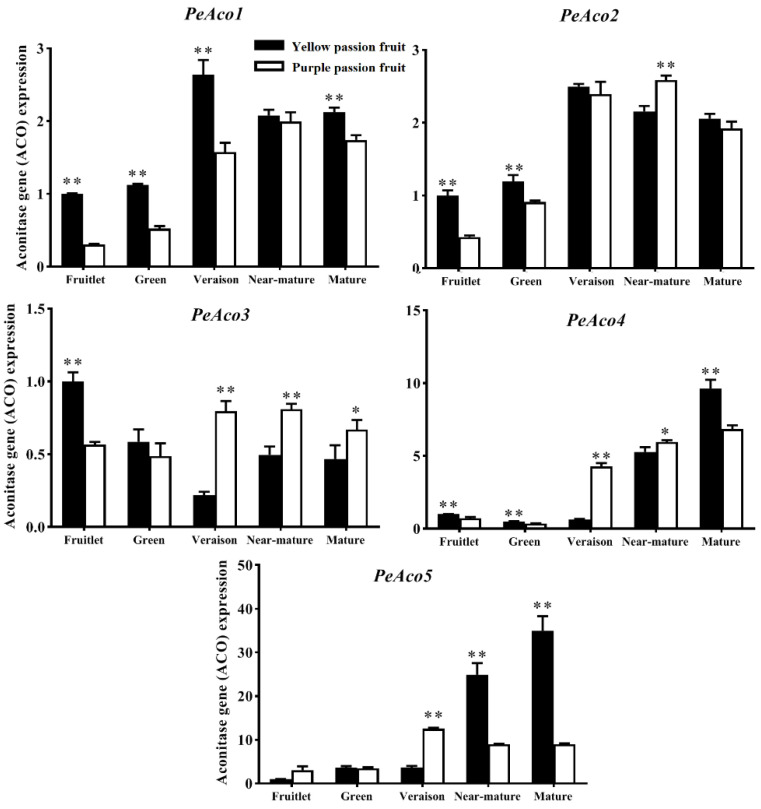
Relative expression of *PeACO* genes in two passion fruit cultivars, (i.e., ‘yellow passion fruit’ and ‘purple passion fruit’) during fruit growth and development. Vertical bars indicate means ± SD (*n* = 3). The * and ** represent significance at *p* ≤ 0.05 and *p* ≤ 0.01, respectively, according to Student’s *t*-test.

ACO having a negative correlation with fruit acidity is possibly a key enzyme that plays a significant role in citric acid degradation [14]. Five genes (*ACO1, ACO2, ACO3, ACO4* and *ACO5*) encoding ACO were confirmed in the passion fruit transcriptome data. Change patterns of *PeACO1, ACO2, ACO4* and *ACO5* genes in both cultivars were almost similar throughout fruit development (Figure 6). The expression level of *ACO* genes was relatively low at early growth stage which paralleled with the high level of citric acid contents. Subsequently, the same genes continually increased and remained constant at relatively high levels from the veraison stage to mature stage. Such pattern accelerated the degradation of citric acid content.

**Figure 7:**
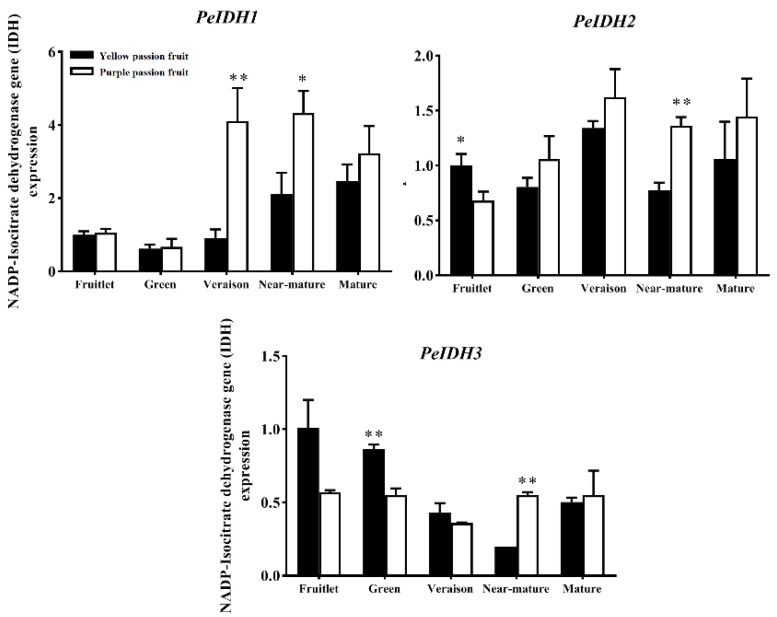
Relative expression of *PeIDH* genes in two passion fruit cultivars, (i.e., ‘yellow passion fruit’ and ‘purple passion fruit’) during fruit growth and development. Vertical bars indicate means ± SD (*n* = 3). The * and ** represent significance at *p* ≤ 0.05 and *p* ≤ 0.01, respectively, according to Student’s *t*-test.

Isocitrate dehydrogenase (IDH) is the key enzyme responsible for citrate catabolism. The passion fruit transcriptome databases and PCR confirmation indicated the presence of at least three *NADP-IDH* (cytoplasmic type) genes in passion fruit genome. The PCR confirmation indicated that the expression pattern of *PeIDH* genes is different among both varieties with fruit growth and development (Figure 7). There was no significant difference (*p* ≤ 0.05) in *PeIDH*1 transcript level between both cultivars at early growth stage, while at mature stage, ‘yellow passion fruit’ showed lower transcript level the ‘purple passion fruit’. Differently from *PeIDH*1, the transcript levels of the *PeIDH*2 and *PeIDH*3 genes were significantly higher in ‘yellow passion fruit’ than ‘purple passion fruit’ from the fruitlet stage to green stage, whereas at late maturity stages (veraison-mature), the expression level of same genes were significantly lower in ‘yellow passion fruit’ than ‘purple passion fruit’.

### 2.5. Correlation Analysis

**Figure 8:**
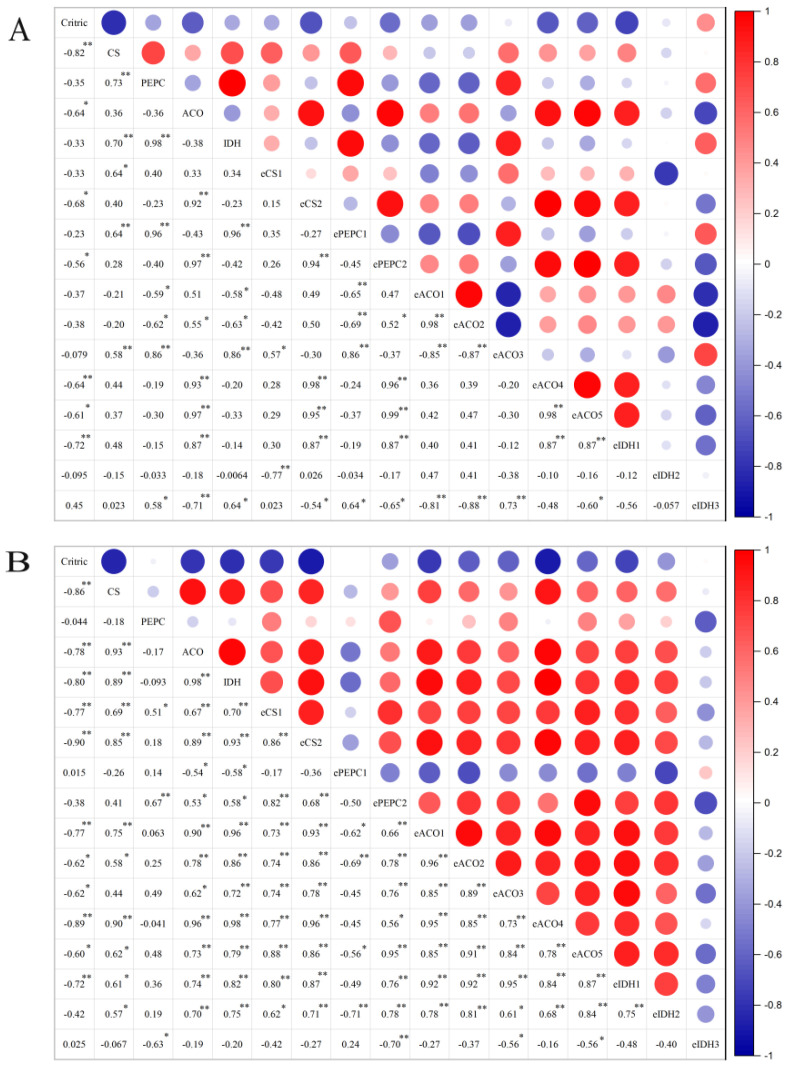
The correlation analysis of citric acid contents, enzymatic activities and expressions of genes involved in citric acid metabolism during fruit development of two passion fruit cultivars, (i.e., ‘yellow passion fruit’ and ‘purple passion fruit’). The * and ** represent significance at *p* ≤ 0.05 and *p* ≤ 0.01, respectively, following Pearson (*n*) method.

The correlation analysis of citric acid contents, enzymatic activities and expressions of genes involved in citric acid metabolism during fruit development of yellow and purple passion fruits was done. The strong negative correlations were observed among CS, ACO, IDH and citric acid content. The negative correlation between CS and ACO was found in both cultivars (Figure 8). In yellow passion fruits, citric acid content was significantly (*p* ≤ 0.05) negatively correlated with the expression of *CS2*, *PEPC2*, *ACO*4-5 and *IDH1* genes, while its correlations with *CS1*, *PEPC*1, *ACO*1-3 and *IDH*2-3 genes were non-significant (*p* ≤ 0.05) (Figure 8A). However, citric acid content of ‘purple passion fruit’ was significantly (*p* ≤ 0.05) negatively correlated with the expressions of *CS1-2, ACO*1-5 and *IDH*1 genes, and its correlations with *PEPC*1-2 and *IDH*2-3 were found non-significant (*p* ≤ 0.05) (Figure 8B). These results suggest that there might be different mechanisms of acid metabolism in both cultivars, leading to the same genes in the citrate metabolism-related enzymes gene family, which play different functions and timeliness in the organic acid metabolism. Furthermore, there were also significant correlations among different enzymatic activities or genetic expressions related to citric acid, and they were synergistically involved in the metabolism of citric acid.

## 3. Discussion

After fruit set initiation, fruit weight of passion fruit increases in a sigmoid fashion [22,23]. In current study, the fresh weight of ‘yellow passion fruit’ showed a typical single sigmoid growth curve, whereas in case of ‘purple passion fruit’, it increased rapidly from the fruitlet stage to veraison stage and then slightly declined until fruit ripening. The growth rate of ‘purple passion fruit’ was faster compared with ‘yellow passion fruit’, making sigmoid curve not obvious. Rapid enhancement in fruit shape index and weight during initial stage could be due to fast cell differentiation and cell enlargement, and subsequent decline towards maturation might be due to loss of water from fruits through transpiration [24]. Titratable acidity of passion fruits increased rapidly at early growth stage and then decreased as the fruit matured, while TSS steadily increased with fruit development [22,23]. The same trend regarding TA and TSS was found for both passion fruit cultivars in our study. It is worth noting that the TA content of ‘yellow passion fruit’ was significantly lower than that of ‘purple passion fruit’. However, the sugar-acid ratio (TSS/TA) of the former is significantly higher than that of the latter, indicating that acid is the main factor affecting the flavor of the fruit.

The organic acids content in fruits is one of the important indicators affecting fruit quality. Fruit quality is also affected by the acid-sugar ratio, while the acid-sugar ratio is mainly determined by acids content [25,26,27]. Organic acid usually accumulates at the early stages of fruit development, and its decrease is caused by enhanced basic metabolism and synthesis of sugar or secondary compounds in the late stage of fruit maturity [28,29,30]. In current study, citric acid content of yellow and purple passion fruits reached a peak at green stage, which were 4720.95 mg·100 g^−1^ FW and 5170.87 mg·100 g^−1^ FW, accounting for 96.73% and 92.53% of the total organic acids, respectively. These results confirm that citric acid is the main organic acid in both cultivars, likely affecting fruit taste [31]. The decrease in citric acid contents of both cultivars during maturation was in agreement with the general observation in other fruits such as, navel orange [32], pear [33] and apple [34]. Additionally, citric acid contents of ‘yellow passion fruit’ experienced a sharp decrease during fruit maturation, while the decline rate of ‘purple passion fruit’ was relatively gentle, making the fruits very sour at maturity.

Citric acid is an intermediate in the TCA cycle, and the synthesis and degradation of citric acid is closely related to the TCA cycle [35]. The changes of citric acid content are consistent with the activities of CS, PEPC and Cyt-Aco enzymes [36], indicating that these are the important enzymes which affect the citric acid metabolism [37]. In order to validate the regulation of citrate contents by organic acid metabolism-related enzymes, the activities of PEPC, CS, Cyt-ACO and Cyt-IDH were observed. The activity of PEPC in ‘yellow passion fruit’ decreased at the early stages of fruit development and increased at the later stages, whereas the activity of same enzyme in ‘purple passion fruit’ first decreased quickly and then increased, followed by rapid decrease until maturity. This was not in accordance with studies conducted on loquat [15], pineapple [38] and plum [30], i.e., that activity of PEPC increased at the early stages of fruit and then decreased during the maturity period, implying that the regulatory effect of PEPC on organic acid metabolism in fruits varies with species. Moreover, citric acid contents of both cultivars did not correlate with PEPC activity through fruit development, which means PEPC activity does not have a decisive effect on the organic acid accumulation in passion fruit.

Citrate synthase has a positive relation with citric acid accumulation in fruits [39,40]. Our results showed that the activity of CS in both cultivars decreased from fruitlet stage to green stage and then increased at later stages. Such trends were opposite to the accumulation trends of citric acid, and CS activity was negatively correlated with the citric acid contents. This phenomenon explained that the high citric acid concentration decreased CS activity by feedback inhibition in the early stages of fruit development, but in the later stage of fruit development, low citric acid content was not enough to maintain normal energy metabolism, thus enhancing the CS activity. This substrate inhibitory effect was also observed in plum [30] and tomato [41].

Evidence showed that ACO and IDH may play a significant role in decreasing citric acid concentrations during a fruits’ maturation including in orange [21], pear [25] and peach [42]. Our results showed the citric acid concentration in both cultivars decreased linearly with increasing Cyt-ACO and Cyt-IDH activity at the later stages of fruit development. The difference is that citric acid concentration in ‘purple passion fruit’ was significantly negatively correlated with Cyt-ACO and Cyt-IDH activity, whereas in ‘yellow passion fruit’, the correlations between the activity of Cyt-IDH and citric acid content were not significant, which indicated that Cyt-IDH does not have a decisive effect on the organic acid degradation in the fruits of ‘yellow passion fruit’. In addition, it is noteworthy that citric acid content of ‘purple passion fruit’ was significantly (*p* ≤ 0.05) greater than that of ‘yellow passion fruit’ throughout fruit development. Combined activities of CS, Cyt-ACO and Cyt-IDH in the Aco-GABA pathway were significantly higher in ‘purple passion fruit’ than ‘yellow passion fruit’. These results implied that ‘purple passion fruit’ had higher abilities for citrate biosynthesis and utilization compared with ‘yellow passion fruit’.

The changes in activities of PEPC, CS, ACO and IDH were regulated by the corresponding genes. The present study showed that citric acid content of ‘yellow passion fruit’ was significantly correlated with the expressions of *CS2*, *PEPC2*, *ACO4*, *ACO5* and *IDH1* genes, whereas *CS1-2, ACO1*, *ACO2, ACO3, ACO4, ACO5* and *IDH1* genes correlated with ‘purple passion fruit’. This phenomenon (same gene has different expression patterns in different varieties) was also found in orange [21]. The strong correlations between citric acid contents and expression level of *CS2, ACO4, ACO5* and *IDH1* genes in both yellow and purple passion fruit were discovered. Interestingly, expression level of *CS2, ACO4, ACO5* and *IDH1* genes of ‘purple passion fruit’ was significantly (*p* ≤ 0.05) greater than that of ‘yellow passion fruit’ at the late stage of fruit development. This is consistent with enzymatic activity results that activities of CS, Cyt-ACO and Cyt-IDH were significantly higher in ‘purple passion fruit’ than that of ‘yellow passion fruit’ during the maturity period. Therefore, we proposed that differences in the expression of genes encoding the CS, Cyt-ACO and Cyt-IDH contributed substantially to the observed differences in organic acid accumulation of both cultivars.

## 4. Materials and Methods

### 4.1. Plant Material and Fruit Quality Evaluation

**Figure 9:**
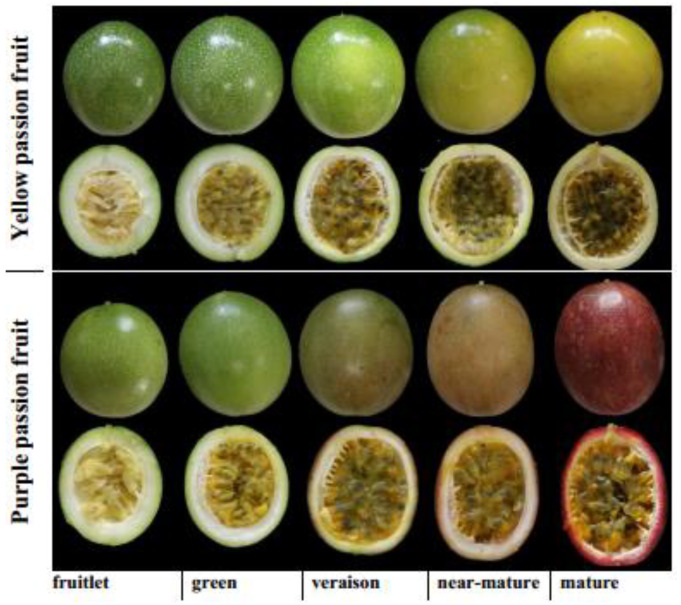
Developmental stages of yellow and purple *Passiflora edulis* Sims.

The fruits from two passion fruit cultivars i.e., yellow and purple were collected from a commercial orchard located at Longyan, Fujian, China. Thirty fruits (five fruits per plant) having uniform size were collected from six different plants of each cultivar at five developmental stages (i.e., fruitlet, green, veraison, near-mature and mature) (Figure 9). Fruit size and color were measured as parameters to monitor the developmental stages. Fruit weight, total soluble solids (TSS) and titratable acidity (TA) were immediately measured after sampling using 15 fruits from each cultivar, and the remaining fruits were carefully dissected into pulp, skin and seeds. Then, pulp was treated with liquid nitrogen and stored at −80 °C until further analysis. Fruit weight was measured with digital weighing balance (MJ-W176P, Panasonic, Japan), whereas TSS was measured using a digital hand-held refractometer (PR101-α, Atago, Japan). To measure TA, fruit juice was titrated with 0.1 M NaOH to the end point at pH 8.2 [39].

### 4.2. Extraction and Determination of Organic Acids

Organic acids were extracted as described by Nour et al. [43], with some modifications. The passion fruit was cut into halves and squeezed to obtain juice. The pulp and seeds were removed by passing through three layers of gauze cloth. After the centrifugation at 4000 rpm for 15 min, the juice supernatant was 25 times diluted and filtered through MF-Millipore™ Membrane Filter (Cat. No. GSWP04700, 0.22 µm pore size). Organic acids were analyzed by ultra-performance liquid chromatography (UPLC). A 10 µL elute sample was injected into an Acquity UPLC HSS T3 column (1.8 µm particle size, 2.1 mm × 100 mm). The flow rate was 0.2 mL min^−1^ using 0.025% H_3_ PO_4_ solution as the solvent. Organic acids were detected at 210 nm, while column temperature was 30 °C. A Waters 2996 diode array detector (Waters Corporation, Milford, MA, USA) was used to detect the eluted peaks. The contents of individual organic acids were calculated using calibration curve of corresponding standard. All measurements for organic acids were performed with three replicates. The output was expressed in grams per liter of fresh juice (g/L juice). The validation parameters consisted at linearity range, limits of detection and quantification [44]. The peaks were identified by their retention times, comparing the UV–Visible spectra and spiking with standards. Quantification has been done using an external standard curve with five points (Table 1).

### 4.3. Enzymes Extraction and Activity Assay

Phosphoenolpyruvate carboxylase (PEPC), citrate synthase (CS), cytosolic aconitase (Cyt-ACO) and cytosolic isocitrate dehydrogenase (Cyt-IDH) were extracted and measured using the Solarbio enzyme activity kits (Solarbio Life Sciences, Beijing, China) according to the manufacturer’s instructions [36,45].

### 4.4. RNA Extraction and Real-Time Quantitative PCR

Total RNA was extracted from passion fruit pulp using a Total RNA kit (TianGen Biotech, Beijing, China). The quantity and quality of RNA were checked using NanoDrop N-1000 spectrophotometer (NanoDrop Technologies, Wilmington, DE, USA) and agarose gel electrophoresis. Prime Script RT Reagent Kit with a gDNA Eraser (TaKaRa, Dalian, China) was used to synthesize first-strand cDNA from 1 µg of total RNA. Real-time qPCR analysis was carried out using high-performance real-time PCR (LightCycler^®^ 96, Roche Applied Science, Penzberg, Germany). The primers for qRT-PCR were designed by Primer-blast and are listed in Table 2.

The reaction mixture contained 10 μL 2 × RealStar Green Fast Mixture (GenStar, Bejing, China), 1 µL cDNA, 0.25 µM of each primer, and water was added to make a final volume of 20 µL. Cycling conditions were as follows: 95 °C for 2 min, 40 cycles of 95 ℃ for 5 s and 60 °C for 30 s. The 60 S ribosomal protein was used as an internal control, and the relative gene expression was calculated using the 2^−ΔΔct^ method [46]. Three independent biological replicates were analyzed for each sample.

### 4.5. Statistical Analysis

Collected data were subjected to one-way analysis of variance (ANOVA) using GraphPad Prism 8.0.1 (https://www.graphpad.com/scientific-software/prism/, accessed on 8 December 2020). Comparison between ‘yellow’ and ‘purple’ passion fruit for each developmental stage was performed using Student’s *t*-test. Correlation coefficient values were determined with Pearson (*n*) method using R software package (http://www.rproject.org/, accessed on 12 January 2021).

## 5. Conclusions

The results of current study suggest that citric acid is the predominant acid among all other observed organic acids in both cultivars of passion fruit. The dynamics of the citric acid contents measured in the current study were not solely controlled by a single enzyme but were regulated by the integrated activity of different enzymes such as CS, Cyt-Aco and Cyt-IDH. Among them, ACO in the cytosol (cyt-ACO) played a key role in the reduction of citric acid during growth and maturation. These results provided new insight into the characteristics of organic acid metabolism and a valuable resource for future research on molecular breeding in passion fruit.

## Figures and Tables

**Table 1:** Validation parameters for the liquid chromatography method.

Acid	Linearity (*r*^2^)	Slope (y)	Response (Sy)	Sy/y	LOD * (µg·mL^−1^)	LOQ ** (µg·mL^−1^)
Tartaric	0.9992	5034.58	9870.7	1.96	6.46	19.6
Ascorbic	0.9998	8025.61	6625.4	0.82	2.72	8.25
Citric	0.9998	34,189.52	32,649.86	0.95	3.15	9.54
Malic	0.9998	6714	5509.95	0.82	2.7	8.2
Lactic	0.9996	4746.69	6666.9	1.4	4.63	14.04
Aconitic	0.9999	2918.31	2134.37	0.73	2.41	7.31

* Limit of detection; ** Limit of quantification.

**Table 2:** Sequences of 12 Primer Pairs for qRT-PCR Analysis.

Primer Name	Forward Primer (5′-3′)	Reverse Primer (5′-3′)
*PEPC1*	GCTGGGATCGAGGATATGGC	CGAACTCTGTGGTGGGTCTC
*PEPC2*	CTGGTAAAGATGCAGGGCGA	CCTCTTCCAACAGTCCCACC
*Aco1*	GCTGAAACACTTGGCTTGACT	GTCTGTGACGACTGCAACATC
*Aco2*	TACGCATGGGAACCCACATC	TCCGAAGTTGAGCAAGCAGT
*Aco3*	AGGTCCACTGCTTTTGGGAG	CACATCCTCACCAGGCTTGA
*Aco4*	TGGCACGGTTGACATTGACT	CACGCTTGATTGCACAACCT
*Aco5*	TTGGCAGACAGAGCTACCAT	CTCTCTCAATCTGGGGCTCAC
*CS1*	TCACTGTTCTTTTTGGCGTGT	AGCCACTCCATCGTTACACT
*CS2*	GTTGCTTTGGAGAAGGCTGC	GTGATTCTCGCCAATGTGCC
*IDH1*	GCTGAAGCAGCTCATGGAAC	TCCCCGATTCAACGACTCTG
*IDH2*	GTTTGAGGCCGCTGGGATA	TCACTTTGCACGTCCCCATC
*IDH3*	CCACAGGGCAAAGTTGGATGA	TGCTCTCAGTTCATCAGCGAC
